# Characteristics of Postmarketing Studies for Vaccines Approved by the US Food and Drug Administration, 2006-2020

**DOI:** 10.1001/jamanetworkopen.2021.8530

**Published:** 2021-04-30

**Authors:** Osman Moneer, ChangWon C. Lee, Jerry Avorn, Aaron S. Kesselheim

**Affiliations:** 1 Program on Regulation, Therapeutics and Law (PORTAL), Division of Pharmacoepidemiology and Pharmacoeconomics, Department of Medicine, Brigham and Women’s Hospital, Harvard Medical School, Boston, Massachusetts; 2 Yale School of Medicine, Yale University, New Haven, Connecticut

## Abstract

This cross-sectional study uses data from the US Food and Drug Administration (FDA) to review the postmarketing requirements and commitments attached to new vaccines approved for use in the past 15 years in the United States.

## Introduction

After the rapid authorization by the US Food and Drug Administration (FDA) of 3 COVID-19 vaccines based on preapproval trials demonstrating favorable benefit-risk profiles, continued monitoring and testing are needed to further clarify their safety.^1^ Once approved, medical products are often subject to postmarketing commitments (PMCs) and postmarketing requirements (PMRs).^2^ Postmarketing commitments are promises made by the manufacturer on FDA approval regarding postapproval testing that it will perform. By contrast, the law requires PMRs to be completed; otherwise, the FDA can institute penalties or remove the product from the market.^3^

Among the 110 new drugs approved between January 1, 2009, and December 31, 2012, 97 (88%) had at least 1 PMR.^4^ We conducted a cross-sectional study to review the characteristics of PMCs and PMRs attached to new vaccines approved in the past 15 years.^5^

## Methods

In this cross-sectional study, we gathered FDA approval letters to identify all PMRs and PMCs for vaccines first approved in the United States between January 1, 2006, and December 31, 2020. We abstracted information using the FDA’s Postmarketing Requirements and Commitments database (updated October 20, 2020), and recorded the most recent study status, original approval date, and status update date. For PMRs, we recorded the formal legal authority under which they were imposed: Pediatric Research Equity Act of 2003 (studies in children), Accelerated Approval Program (confirmatory studies for approvals based on surrogate measures characterized by substantial uncertainty), and section 505(o)(3) of the Federal Food, Drug, and Cosmetic Act (studies required to assess serious risks). On the basis of previous work, each PMR and PMC was further categorized by study type.^4^ This study used public, nonidentifiable data that do not constitute human subject research (45 CFR 46.102) and was not submitted for institutional review board approval. This study followed the Strengthening the Reporting of Observational Studies in Epidemiology (STROBE) reporting guideline for cross-sectional studies.

The FDA reports study status as either open (pending, ongoing, delayed, terminated, or submitted) or closed (in which the obligation was fulfilled or the manufacturer was released from its obligation). Because the FDA database expunges closed studies after 1 year, we supplemented our search using archived previous versions of the database, product supplemental approval letters, and manufacturer status reports. All analyses were performed using Microsoft Excel version 16.46 (Microsoft Corp).

## Results

The 35 approved vaccines had 72 PMCs and 56 PMRs, a median of 4 PMCs or PMRs (interquartile range [IQR], 2-5 PMCs or PMRs) per vaccine. New vaccine approvals ranged from 1 to 4 per year, and only 8 of the 35 vaccines (22.9%) were approved in the past 5 years (Table 1). All vaccines except one were approved with at least 1 PMR or PMC. Postmarketing commitments constituted more than half of postmarketing studies (72 of 128 studies [56.3%]). Among the total PMCs and PMRs, 45 PMRs (35.1%) were mandated under the Pediatric Research Equity Act of 2003, 8 (6.3%) as a condition of accelerated approval, and 3 (2.3%) under section 505(o)(3) authority.

**Table 1.** New Vaccine PMRs and PMCs by Year

Year	No. (%)		PMRs or PMCs per product
	Vaccines approved	PMRs or PMCs instituted	
2006	4 (11.4)	15 (11.7)	3. 8
2007	3 (8.6)	16 (12.5)	5.3
2008	3 (8.6)	3 (2.3)	1.0
2009	4 (11.4)	17 (13.3)	4.3
2010	2 (5.7)	14 (10.9)	7.0
2011	1 (2.9)	3 (2.3)	3.0
2012	2 (5.7)	7 (5.5)	3.5
2013	2 (5.7)	9 (7.0)	4.5
2014	2 (5.7)	12 (9.4)	6.0
2015	3 (8.6)	11 (8.6)	3.7
2016	1 (2.9)	2 (1.6)	2.0
2017	2 (5.7)	5 (3.9)	2.5
2018	1 (2.9)	0	0
2019	3 (8.6)	6 (4.7)	2.0
2020	2 (5.7)	8 (6.3)	4.0
All years	35	128	NA

Abbreviations: NA, not applicable; PMCs, postmarketing commitments; PMRs, postmarketing requirements.

Most PMRs and PMCs were for new studies (104 of 128 studies [81.3%]) with specifications including prospective trials and retrospective observational studies. The remaining sample concerned the completion or submission of study results (16 of 128 studies [12.5%]) or the analysis or follow-up from existing studies (8 of 128 studies [6.3%]).

The median time from vaccine approval to PMC or PMR completion (fulfilled or released) was 50 months (IQR, 34-92 months)]. Approximately half of the studies (62 of 128 [48.4%]) were fulfilled, whereas approximately one-tenth (14 of 128 [10.9%]) were released (Table 2). Postmarketing commitments had a 54.2% rate of being fulfilled (39 of 72) compared with 41.1% for all PMRs (23 of 56). The requirement to conduct new prospective cohort studies, registries, and clinical trials was the most common form of postapproval research (85 of 128 [66.4%]) but was the least likely to be categorized as fulfilled (36.4% vs a mean of 71.4% [range, 57.8%-93.8%]) for other study types (eg, new retrospective observational studies).

**Table 2.** Vaccine Postmarketing Study Status by Legal Authority and Study Category

Legal authority and study category	Study status, No. (%)^a^							Total
	Pending	Ongoing	Delayed	Submitted	Fulfilled	Released	Missing	
All	8 (6.3)	18 (14.0)	3 (2.3)	13 (10.2)	62 (48.4)	14 (11.0)	10 (7.8)	128
Legal authority								
21 CFR 601.70^b^	5 (6.9)	7 (9.7)	2 (2.7)	2 (2.7)	39 (54.2)	8 (11.1)	9 (12.5)	72
Pediatric Research Equity Act of 2003	3 (6.6)	9 (20.0)	0	9 (20.0)	18 (40.0)	6 (13.3)	0	45
Section 505(o)(3)^c^	0	1 (33.3)		0	1 (33.3)	0	1 (33.3)	3
Accelerated approval	0	1 (12.5)	1 (12.5)	2 (25.0)	4 (50.0)	0	0	8
Study category								
New retrospective observational studies	0	0	0	1 (5.3)	11 (57.8)	3 (15.7)	4 (21.0)	19
New prospective cohort studies, registries, and clinical trials	8 (9.4)	16 (18.8)	3 (3.5)	10 (11.8)	31 (36.4)	11 (12.9)	6 (7.1)	85
Analyze or follow up from observational studies, registries, or clinical trials (and other flexible requirements)	0	1 (12.5)	0	2 (25.0)	5 (62.5)	0	0	8
Complete or submit results from ongoing prospective cohort studies, registries, and clinical trials	0	1 (6.3)	0	0	15 (93.8)	0	0	16

^a^ Percentages may not total 100% because of rounding.

^b^ 21 CFR §601.70 refers to the legal authority for postmarketing commitments.

^c^ Section 505(o)(3) refers to the Postmarketing Studies and Clinical Trials section of the Federal Food, Drug, and Cosmetic Act (21 USC §355(o)(3)).

## Discussion

Vaccines are commonly approved by the FDA with PMCs and PMRs, but only approximately half were considered fulfilled in 2020, and the time to completion was a median of more than 4 years. One limitation of this study is that not enough time has elapsed to observe completion rates of PMCs and PMRs for recently approved vaccines, although only 8 of the 35 vaccines (22.9%) in the sample were approved in the past 5 years.

Given the track record of lengthy time to completion for postmarketing studies of new vaccines, it will be important to ensure that any PMCs or PMRs associated with COVID-19 vaccines are fulfilled in a timely fashion.
